# Psychosocial determinants associated with healthcare workers’ self-reported compliance with infection prevention and control during the COVID-19 pandemic: a cross-sectional study in Dutch residential care facilities for people with intellectual and developmental disabilities

**DOI:** 10.1186/s12889-023-16912-0

**Published:** 2023-10-19

**Authors:** Famke Houben, Casper DJ den Heijer, Nicole HTM Dukers-Muijrers, Claudia Smeets-Peels, Christian JPA Hoebe

**Affiliations:** 1 Department of Social Medicine, Care and Public Health Research Institute (CAPHRI), Faculty of Health, Medicine and Life Sciences, Maastricht University, P.O. Box 616, Maastricht, 6200 MD The Netherlands; 2 Department of Sexual Health, Infectious Diseases and Environmental Health, Living Lab Public Health, South Limburg Public Health Service, P.O. Box 33, Heerlen, 6400 AA The Netherlands; 3 Department of Medical Microbiology, Infectious Diseases and Infection Prevention, Care and Public Health Research Institute (CAPHRI), Faculty of Health, Medicine and Life Sciences, Maastricht University Medical Centre (MUMC+), P.O. Box 5800, Maastricht, 6202 AZ The Netherlands; 4 Department of Health Promotion, Care and Public Health Research Institute (CAPHRI), Faculty of Health, Medicine and Life Sciences, Maastricht University, P.O. Box 616, Maastricht, 6200 MD The Netherlands; 5 Stichting Pergamijn, Mercator 2, Sittard, 6135 KW The Netherlands

**Keywords:** COVID-19, Infection control, Long-term care, Intellectual disability, Developmental disability, Cross-sectional studies

## Abstract

**Background:**

Healthcare workers’ (HCWs) compliance with infection prevention and control (IPC) is crucial to reduce the infection transmission risk. However, HCWs’ compliance with IPC in residential care facilities (RCFs) for people with intellectual and developmental disabilities (IDDs) is known to be suboptimal. Therefore, this study examined sociodemographic and psychosocial determinants associated with IPC non-compliance in this setting, to inform IPC policy and promotion programmes for adequate IPC behaviour.

**Methods:**

An online questionnaire was administered to 285 HCWs from 16 RCFs between March 2021 and March 2022. Determinants associated with IPC non-compliance were assessed using logistic regression analyses.

**Results:**

Being a *woman* (OR: 3.57; 1.73–7.37), and being a *non-medical professional* were associated with increased odds of non-compliance (social workers, OR: 2.83; 1.65–4.85; behavioural specialists, OR: 6.09; 1.98–18.72). *Perceived inadequate education/training* (aOR: 1.62; 1.15–2.27) and *perceived time constraints/competing priorities* (aOR: 1.43; 1.03–1.98) were also associated with increased odds of non-compliance, independent of sociodemographic variables. In contrast, the belief that the supervisor complies with IPC (*descriptive norm supervisor)* was associated with decreased odds of non-compliance (aOR: 0.60; 0.41–0.88).

**Conclusions:**

To improve IPC in disability care settings, the implementation of tailored and structural IPC education and training programmes (e.g., on-the-job training) is recommended to increase HCWs’ capabilities and bridge the IPC compliance gap between medical and non-medical professionals. In addition, role models, particularly supervisors, are crucial for promoting IPC behaviour. Facilities should create a culture of IPC compliance by norm setting, acting on, and modelling IPC behaviours at all levels of the organisation (management, medical, and non-medical staff).

## Background

Healthcare workers (HCWs) play a key role in controlling outbreaks and preventing infections in care institutions [1]. HCWs should follow appropriate infection prevention and control (IPC) practices (e.g., hand hygiene and the use of personal protective equipment) to prevent the spread of infectious diseases, including Coronavirus Disease 2019 (COVID-19). Especially in institutional care environments such as residential care facilities (RCFs) for people with intellectual and developmental disabilities (IDDs), the risk of emergence and spread of infections is high [2, 3]. In addition to the high-risk setting, HCWs’ compliance with IPC is important because of the susceptibility of the patient population. Individuals with IDDs are more susceptible to infections due to their underlying medical and physical conditions [4–7].

Despite the importance of HCWs complying with IPC, this compliance has been reported to be suboptimal in RCFs for people with IDDs [8]. Therefore, appropriate behavioural changes among HCWs are necessary. The application of behaviour change theories can be an effective way to improve the compliance of HCWs with IPC practices [9]. By understanding the underlying factors that influence behaviour, it is possible to develop strategies that can encourage HCWs to adopt and maintain IPC practices.

According to behaviour change theories, an individual’s ability and willingness to adopt and maintain a behaviour are affected by a number of psychosocial determinants. At the individual level, cognitive factors, including individuals’ beliefs about the risks and benefits of a behaviour, their attitudes towards a behaviour, and their perceived ability to engage in the behaviour – self-efficacy – can play a role [10–13]. Next to individual determinants, the social environment can influence an individual’s willingness and capacity to adopt a certain behaviour [10, 14]. For instance, social norms can influence behaviour by shaping an individual’s beliefs and expectations about acceptable behaviour in a particular situation. In the context of IPC compliance, previous studies have shown that psychosocial determinants such as attitudes, self-efficacy, and social norms are established factors that influence IPC behaviour among HCWs [15–21].

Besides psychosocial determinants, IPC compliance may differ across sociodemographic factors as age, sex, and occupation. Previous studies in other healthcare settings have demonstrated that adequate IPC practices were more prevalent among younger HCWs [22], whereas other studies have argued that older HCWs reported higher levels of IPC compliance [23]. Furthermore, prior studies have indicated that female HCWs were more likely to comply with IPC [23, 24], whereas other studies did not demonstrate a relationship between age or sex and IPC compliance [25]. Previous studies have also suggested an association between occupation and compliance with IPC, in which non-medical professionals reported lower compliance levels than medical professionals [8, 26–28].

Despite previous efforts in other healthcare settings, little is known about the determinants of IPC compliance among HCWs in disability care. Therefore, this study examined sociodemographic and psychosocial determinants associated with IPC non-compliance among HCWs in RCFs for people with intellectual and developmental disabilities (IDDs), to inform IPC policy and promotion programmes for adequate IPC behaviour.

## Methods

### Study design and setting

A cross-sectional questionnaire study was performed. This study was part of the larger mixed methods study NIEZT (Needs assessment for infection prevention among healthcare professionals outside the hospital) (grant number: 331618). The objective of the NIEZT study was to assess IPC compliance and its determinants among HCWs in Dutch RCFs for people with IDDs.

In the Netherlands, approximately 110,000 individuals with IDDs reside in RCFs [29]. The care setting is characterised by providing care to a range of different clients, including individuals with mild, moderate, severe, and profound intellectual disabilities [30]. Due to the diversity in different client groups, and therefore different care needs, the disability care sector is also characterised by a broad range of different HCWs, including medical professionals (e.g., nurses and physicians), social workers (e.g., personal attendants), and behavioural specialists (e.g., psychologists and therapists).

### Participants

Participants included HCWs from 16 Dutch RCFs for people with IDDs in the southern and western regions of The Netherlands. HCWs who had direct patient contact were considered eligible for the study. Exclusion criteria encompassed managerial or policy-related professionals, such as managers and policy officers, as well as administrative personnel without direct patient contact. We aimed to include HCWs from a variety of occupations and educational backgrounds, in order to identify all relevant determinants of IPC compliance among the broad set of different HCWs in the disability care setting.

### Materials and procedure

We developed an online questionnaire. The first part included questions regarding sociodemographic factors, such as age, sex, and occupation. The second part of the questionnaire assessed the levels of self-reported IPC compliance, which were based on national IPC guidelines of the National Institute for Public Health and the Environment (RIVM) [31, 32]. Additionally, we included questions on psychosocial determinants of IPC compliance.

Prior to distributing the questionnaire to our participants, it was piloted among disability care physicians and reviewed by an infection control professional to ensure its applicability. The experts assessed the questionnaire to be applicable, and only minor modifications (i.e., shortening of the length) were applied.

Convenience sampling was used to recruit participants. A contact person at each umbrella organisation in the disability care sector was contacted by email or telephone, inviting them to voluntarily participate and distribute the questionnaire among their staff members within their organisations in exchange for receiving a facility-specific report on HCWs’ compliance with IPC and its determinants. Information on the study and its aims was provided. After two weeks, a reminder was sent to the contact person in case no responses were received. Responses were collected between March 2021 and March 2022 (during the COVID-19 pandemic: mainly Alpha and Delta variant period).

Informed consent (digitally provided) to participate in this study was obtained from every participant before starting the data collection.

### Measures

#### Outcome

Self-reported IPC compliance was the outcome of interest. IPC compliance was measured by 16 items [31, 32], covering multiple domains of IPC, including hand hygiene, personal hygiene, clothing requirements, use of personal protective equipment, laundry regulations, medical safety procedures, isolation procedures, and antibiotic prescription behaviour. Participants were requested to estimate how frequently they performed the IPC practices in their day-to-day activities using a 5-point Likert scale, with response options ranging from 1 (“never”) to 5 (“always”). HCWs’ compliance was scored ‘1’ (sufficient compliance) if the HCW responded either “always” or “most of the time” (score of 4 or higher on the 5-point Likert scale), otherwise the healthcare worker was scored ‘0’ (inadequate compliance) [33, 34], giving a total possible score range of 0–16. Finally, a total compliance score for all IPC practices was calculated by dividing the number of IPC practices marked as compliant by the total number of required practices (for the specific occupation) [1, 23]. The total score was expressed in terms of percentages. Subsequently, the total scores were dichotomised. A total compliance score of ≥ 80% was categorised as sufficient compliance, and a compliance score of < 80% as inadequate compliance. This cut-off point was chosen in accordance with previous studies [35, 36]. Detailed information on IPC compliance among HCWs in RCFs for people with IDDs has been described elsewhere [8].

In this study, inadequate compliance was operationalised as non-compliance to improve readability. However, it is important to note that compliance is a gradient that ranges from fully sufficient to complete non-compliance.

#### Determinants

##### Sociodemographic variables (non-modifiable determinants)

Age, sex, and occupation were included as sociodemographic variables. Professional group was computed based on the occupation and associated job descriptions, tasks, and responsibilities [8].

##### Psychosocial determinants (modifiable determinants)

The included items measuring psychosocial determinants comprised a range of items related to attitudes, self-efficacy, and social norms. The questionnaire included 16 individual 1-item statements. Individual statements were used to gain a more detailed understanding of participants’ beliefs, which allows for more targeted interventions [37, 38]. The included psychosocial determinants were selected based on findings from our qualitative study [39] and concepts derived from the Health Belief Model, Theory of Planned Behaviour (including the attitude, social influence, and self-efficacy [ASE]-model), Theory of Interpersonal Behaviour, and the Social Cognitive Theory [10–13], and adapted from previously developed instruments [20, 33]. Participants were asked to rate their level of agreement with each statement on a 5-point Likert scale ranging from 1 (“strongly disagree”) to 5 (“strongly agree”). Table 1 provides the measurement of psychosocial determinants.

**Table 1:** Measurement of psychosocial determinants

Psychosocial determinants	Item
Attitudes	
Perceived importance IPC	*I consider complying with IPC to be important.*
Resistance towards protocols/guidelines	*Working according to protocols/guidelines evokes resistance in me.*
Perceived interference with the professional-client relationship	*Complying with IPC interferes with the professional-client relationship.*
Perceived effort	*Complying with IPC requires a lot of effort.*
Perceived time investment	*Complying with IPC takes a lot of time.*
Perceived risk for clients	*I expect the client has a high risk of infection.*
Outcome expectation	*I expect that infections can be prevented if I comply with IPC.*
Self-efficacy	
General self-efficacy	*I feel confident in my ability to comply with IPC.*
Perceived inadequate education/training	*I am not educated/trained to comply with IPC.*
Perceived knowledge	*I have sufficient knowledge to comply with IPC.*
Perceived skills	*I have sufficient skills to comply with IPC.*
Perceived time constraints/competing priorities	*Due to busyness with other tasks, I often forget to comply with IPC.*
Social norms	
Descriptive norm colleagues	*Colleagues comply with IPC.*
Descriptive norm supervisor	*Supervisor complies with IPC.*
Injunctive norm colleagues	*My colleagues think I should comply with IPC.*
Injunctive norm supervisor	*My supervisor thinks I should comply with IPC.*

*Abbreviations.* IPC = infection prevention and control

### Statistical analyses

For this study, only complete questionnaires were included. Participants who did not provide direct patient care (e.g., managers, policy-related professionals, and administrative personnel) were excluded (n = 38). Secondly, descriptive statistics were used to represent participant characteristics. In addition, descriptive statistics were provided for the distribution of answers for the psychosocial determinants. To assess the associations between determinants and IPC non-compliance, we used binary logistic regression. Firstly, associations between IPC non-compliance and all sociodemographic variables were assessed using univariable logistic regression analyses. Secondly, associations between IPC non-compliance and psychosocial determinants were assessed using multivariable logistic regression analyses, adjusted for sociodemographic variables. As we aimed to examine all relevant psychosocial determinants of IPC non-compliance, separate multivariable models were computed for each individual psychosocial determinant. To identify factors that could be targeted in interventions, it is important to focus on factors that have potential room for improvement [40]. Therefore, items with high (dis)agreement (i.e., little contrast) – more than 90% of HCWs scored strongly (dis)agree or (dis)agree – were excluded from our models, as they are less likely to influence behavioural change. The resulting associations were reported using (adjusted) odds ratios (ORs) with 95% confidence intervals (CIs). For the psychosocial determinants, the odds of non-compliance were expressed for each point increase in psychosocial determinant scores. Moreover, interaction terms between each psychosocial determinant and professional group were added to the regression models to assess whether associations between psychosocial determinants and IPC non-compliance varied between different professional groups. A *p*-value < 0.1 was considered relevant to indicate effect modification. In case of effect modification, the associations between psychosocial determinants and IPC non-compliance were examined per professional group. Lastly, sensitivity analyses were conducted to assess the cut-off point for sufficient compliance by repeating the analysis with different operationalisations (cut-off points: 65%, 70%, and 75%). All analyses were performed using IBM SPSS Statistics version 27, and a *p*-value < 0.05 was considered as statistically significant.

## Results

### Study population

In total, 323 complete questionnaire responses were received, of which 285 (88.2%) met the inclusion criteria. Out of the 20 facilities approached, responses were obtained from participants from 16 facilities (80%). Non-participation reasons were time constraints and staff shortages arising from the significant burden of COVID-19 cases and outbreaks in the facilities. Table 2 presents the participant characteristics. Most participants were women (87.4%), with a mean age of 43 (± 12.5 years). Half of the participants were social workers (55.1%).

**Table 2:** Participant characteristics (N = 285)

	N (%)/M (SD)
*Sex*	
Man	35 (12.3%)
Woman	250 (87.7%)
*Age*	43 (12.5)
*Professional group*	
Medical professionals^a^	97 (34.0%)
Social workers^b^	157 (55.1%)
Behavioural specialists^c^	31 (10.9%)

^a^ Medical professionals include physicians, nurses, medical assistants, nursing assistants, and paramedical professionals with direct physical contact with clients (e.g., physiotherapists)

^b^ Social workers include for instance personal care attendants and other support staff who provide assistance to clients with daily tasks (including personal hygiene facilitation, and mobility support)

^c^ Behavioural specialists include psychologists, behavioural scientists, remedial educationalists, and coaches/therapists

### Distribution of response scores for psychosocial determinants

**Fig. 1:**
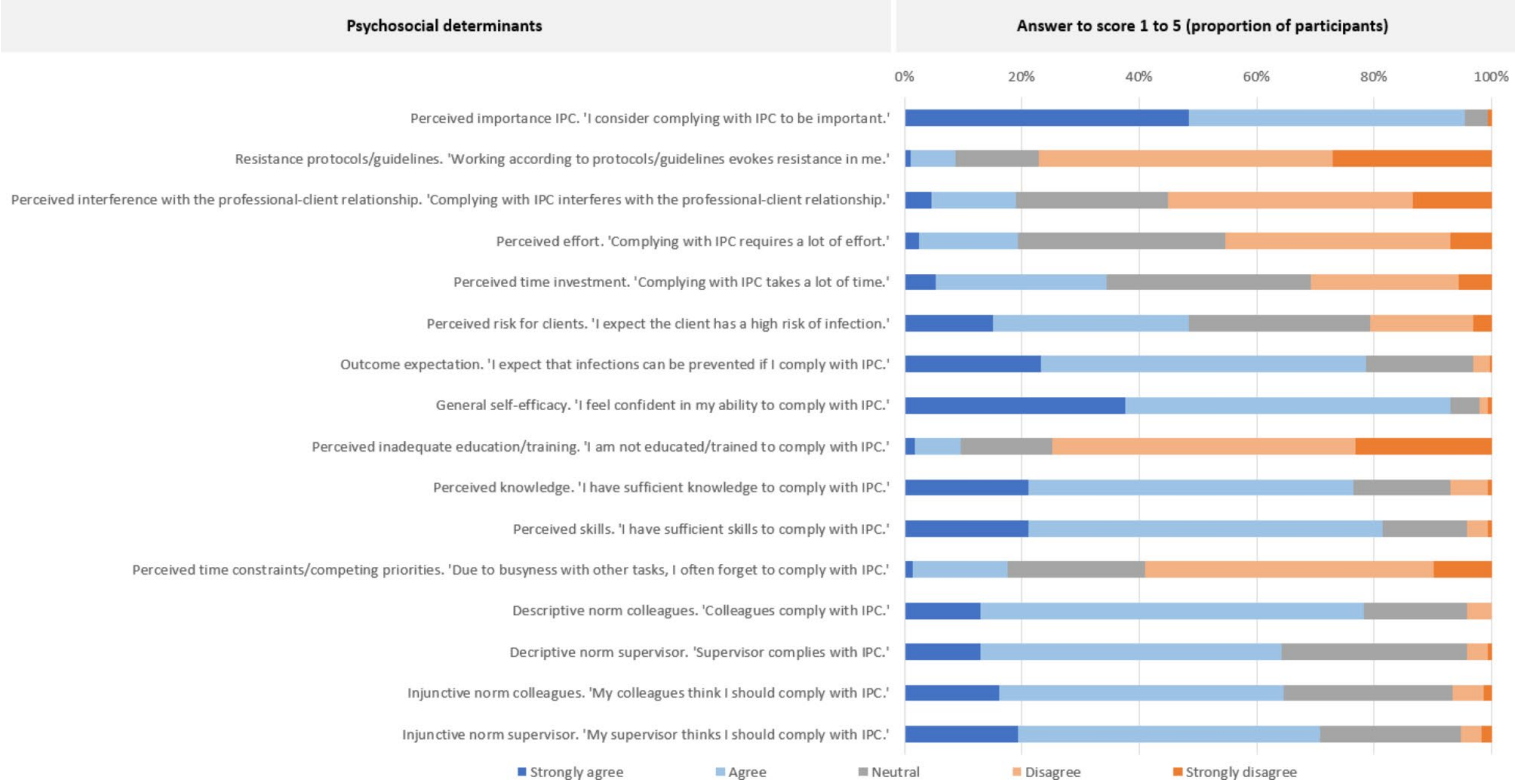
Distribution of responses to the items regarding psychosocial determinants

Figure 1 presents the distribution of response scores (1 to 5) for each psychosocial determinant. Regarding attitudes, 95.4% (n = 272) considered complying with IPC to be important. In addition, only 8.8% (n = 25) of participants reported that working according to protocols/guidelines evokes resistance. Regarding perceived interference with the professional-client relationship, 18.9% (n = 54) reported to strongly agree or agree. Furthermore, 19.3% (n = 55) reported that complying with IPC requires a lot of effort, and 34.4% (n = 98) reported that complying with IPC takes a lot of time. Moreover, 48.4% (n = 138) of participants expected the client to have a high risk of infection. Regarding outcome expectation, 78.6% (n = 224) expected that infections can be prevented when complying with IPC. Regarding general self-efficacy, 93.0% (n = 265) reported feeling confident about their ability to comply with IPC. In addition, 9.5% (n = 27) of participants reported that they were not educated/trained to comply with IPC. Moreover, 76.5% (n = 218) perceived sufficient knowledge, and 81.4% (n = 232) perceived sufficient skills to comply with IPC. Of the included HCWs, 17.5% (n = 50) reported forgetting to comply with IPC due to busyness with other tasks. Regarding social norms, 78.2% (n = 223) believed that colleagues comply with IPC, and 64.2% (n = 183) believed that the supervisor complies with IPC (descriptive norm). Moreover, 64.6% (n = 184) believed that colleagues think they should comply with IPC, and 70.9% (n = 202) believed that their supervisor thinks they should comply with IPC (injunctive norm).

### Determinants associated with IPC non-compliance

**Fig. 2:**
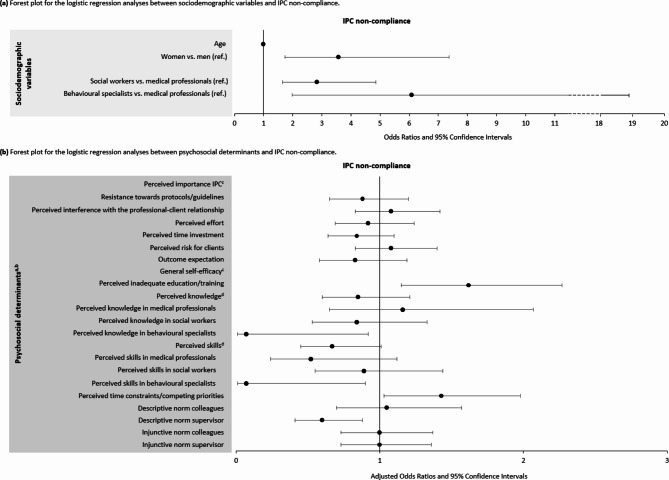
Forest plot showing odds ratios (ORs) and 95% confidence intervals (CIs) for the logistic regression analyses between **a** sociodemographic variables and IPC non-compliance and **b** psychosocial determinants and IPC non-compliance. The circles correspond to the ORs. The error bars (horizontal lines) represent 95% CIs. The vertical line indicates the point of no effect (OR = 1) Abbreviations. IPC = infection prevention and control. Ref = reference category ^a^Analyses for the psychosocial determinants were adjusted for sociodemographic variables ^b^Associations for each point increase in psychosocial determinant scores ^c^Items were not included in the analyses due to high (dis)agreement (i.e., little contrast), > 90% of HCWs scored strongly (dis)agree or (dis)agree ^d^The results of the regression models including interaction terms between ‘perceived knowledge’ and ‘professional group’, and ‘perceived skills’ and ‘professional group’ revealed relevant effect modification (perceived knowledge, *p* = 0.1; perceived skills, *p* = 0.1). Therefore, the associations between these determinants and IPC non-compliance were examined per professional group

To assess the determinants associated with IPC non-compliance, both sociodemographic variables (non-modifiable determinants) and psychosocial determinants (modifiable determinants) were examined. Table 3 presents the results of the logistic regression analyses. Figure 2 provides a visual presentation of the results.

**Table 3:** Logistic regression analysis examining the associations between both sociodemographic and psychosocial determinants and IPC non-compliance

		IPC non-compliance		
Determinants				
	Proportion of participants who strongly agree or agree	OR/aOR (95% CI)		*p*-value
**Sociodemographic variables**				
*Age*		1.00 (0.98–1.02)	0.834	
*Sex*				
Man		Ref.		
Woman		**3.57 (1.73–7.37)**	< 0.001	
*Professional group*				
Medical professionals		Ref.		
Social workers		**2.83 (1.65–4.85)**	< 0.001	
Behavioural specialists		**6.09 (1.98–18.72)**	0.002	
**Psychosocial determinants** ^**a**, **b**^				
Attitudes				
*Perceived importance IPC*	95.4%	NA^c^		
*Resistance towards protocols/guidelines*	8.8%	0.88 (0.65–1.20)		0.421
*Perceived interference with the professional-client relationship*	19%	1.08 (0.83–1.42)		0.559
*Perceived effort*	19.3%	0.92 (0.69–1.24)		0.603
*Perceived time investment*	34.4%	0.84 (0.64–1.10)		0.221
*Perceived risk for clients*	48.4%	1.08 (0.83–1.40)		0.567
*Outcome expectation*	78.6%	0.83 (0.58–1.19)		0.305
Self-efficacy				
*General self-efficacy*	92.9%	NA		
*Perceived inadequate education/training*	9.5%	**1.62 (1.15–2.27)**		0.006
*Perceived knowledge* ^*d*^	76.5%	0.85 (0.60–1.21)		0.364
*Perceived knowledge in medical professionals*	88.6%	1.16 (0.65–2.07)		0.625
*Perceived knowledge in social workers*	71.3%	0.84 (0.53–1.33)		0.459
*Perceived knowledge in behavioural specialists*	64.5%	**0.07 (0.01–0.92)**		0.043
*Perceived skills* ^*d*^	81.5%	0.67 (0.45–1.01)		0.057
*Perceived skills in medical professionals*	93.8%	0.52 (0.24–1.12)		0.096
*Perceived skills in social workers*	77.1%	0.89 (0.55–1.44)		0.636
*Perceived skills in behavioural specialists*	64.5%	**0.07 (0.01–0.90)**		0.042
*Perceived time constraints/competing priorities*	17.5%	**1.43 (1.03–1.98)**		0.032
Social norms				
*Descriptive norm colleagues*	78.3%	1.05 (0.70–1.57)		0.809
*Descriptive norm supervisor*	64.2%	**0.60 (0.41–0.88)**		0.009
*Injunctive norm colleagues*	64.5%	1.00 (0.73–1.37)		0.991
*Injunctive norm supervisor*	70.9%	1.00 (0.73–1.36)		0.974

*Abbreviations.* IPC = infection prevention and control, OR = odds ratio, CI = confidence interval, Ref = reference category, NA = not applicable

^a^Analyses for the psychosocial determinants were adjusted for sociodemographic variables

^b^Associations for each point increase in psychosocial determinant scores

^c^Items were not applicable since they were not included in the analyses due to high (dis)agreement (i.e., little contrast), > 90% of HCWs scored strongly (dis)agree or (dis)agree

^d^The results of the regression models including interaction terms between ‘perceived knowledge’ and ‘professional group’, and ‘perceived skills’ and ‘professional group’ revealed relevant effect modification (perceived knowledge, *p* = 0.1; perceived skills, *p* = 0.1). Therefore, the associations between these determinants and IPC non-compliance were examined per professional group

### Sociodemographic variables associated with IPC non-compliance

For the sociodemographic variables, women had increased odds of non-compliance (OR: 3.57; 1.73–7.37, *p* < 0.001) compared to men. In addition, non-medical professionals were associated with non-compliance; social workers (OR: 2.83; 1.65–4.85, *p* < 0.001) and behavioural specialists (OR: 6.09; 1.98–18.72, *p* = 0.002) had higher odds of non-compliance than medical professionals.

### Psychosocial determinants associated with IPC non-compliance

After adjusting for sociodemographic variables (age, sex, professional group), *perceived inadequate education/training* was associated with increased odds of non-compliance (aOR: 1.62; 1.15–2.27, *p =* 0.006). In addition, *perceived time constraints/competing priorities* was associated with higher odds of non-compliance (aOR: 1.43; 1.03–1.98, *p*  = 0.032). The association between *perceived knowledge* and IPC non-compliance varied for different professional groups (*p* = 0.1, relevant effect modification). Within the group behavioural specialists, *perceived knowledge* was associated with decreased odds of non-compliance (aOR: 0.07; 0.01–0.92, *p* = 0.043). Furthermore, the association between *perceived skills* and IPC non-compliance varied for different professional groups (*p* = 0.1, relevant effect modification). Within the group behavioural specialists, *perceived skills* was significantly associated with decreased odds of non-compliance (aOR:0.07; 0.01–0.90, *p* = 0.042). For all participants, *perceived skills* was borderline significantly associated with decreased odds of non-compliance (aOR: 0.67; 0.45–1.01, *p* = 0.057). *Descriptive norm supervisor* was associated with decreased odds of non-compliance (aOR: 0.60; 0.41–0.88, *p* = 0.009).

### Sensitivity analyses for the cut-off point for sufficient compliance

Sensitivity analyses using different cut-off points (i.e., 65%, 70%, and 75%) for sufficient compliance revealed highly similar results regarding the determinants associated with IPC non-compliance (data not shown).

## Discussion

The examination of determinants associated with HCWs’ compliance with IPC increases the understanding of factors affecting IPC behaviour, which informs the development of intervention programmes for improving compliance. This study assessed sociodemographic and psychosocial determinants of HCWs’ self-reported compliance in residential care facilities (RCFs) for people with intellectual and developmental disabilities (IDDs).

This study showed that the vast majority of included HCWs considered complying with IPC to be important and generally believed they were able to comply with IPC. In addition, fewer than half of the participants perceived clients to be at a high risk of infection, which reveals a potential underestimation of the risk of infection that clients face within RCFs.

Regarding the associations between sociodemographic variables and IPC compliance, our findings demonstrated that non-medical professionals (social workers and behavioural specialists) had increased odds of non-compliance than medical professionals. This finding is consistent with previous studies [8, 26–28], which have indicated that health attendants, personal care workers, and non-clinical staff are more likely to be non-compliant compared to nurses and medical professionals. A potential reason for this may be the difference in professional background [39]. In general, medical professionals are more acquainted with IPC practices, as this is often a part of their training, especially in light of performing medical and nursing procedures. Moreover, the results of this study revealed increased odds of non-compliance in female HCWs compared to their male counterparts. However, careful interpretation of this finding is advised, as previous studies have shown conflicting results. Some studies have suggested that women are more likely to comply with infection control guidelines than men [23, 24], while others have found no significant differences between male and female HCWs [25]. One should note that these previous studies were exclusively conducted among medical professionals. Therefore, caution is needed when comparing these findings with the findings of our study, which included both medical and non-medical professionals. Previous studies conducted in other healthcare settings also suggested an association between years of experience and IPC compliance levels during the COVID-19 pandemic [21, 26]. Overall, these studies indicated that HCWs with more (years of) experience were more likely to comply with IPC practices. In our study, we did not include years of experience as a variable, because of the risk of multicollinearity. Nevertheless, we included age and occupation as covariates, thereby indirectly accounting for the years of experience of HCWs.

Regarding psychosocial determinants, our findings suggested that the self-efficacy items *perceived time constraints/competing priorities* and *perceived inadequate education/training* were independently associated with IPC non-compliance. Previous studies have identified perceived time constraints and competing priorities as important determinants that negatively influence IPC compliance [41, 42]. HCWs often have multiple tasks and responsibilities, and compliance with IPC may sometimes conflict with other tasks or priorities, such as attending to patients’ needs or administrative duties. Furthermore, inadequate education or training is commonly reported to be negatively associated with IPC compliance [42, 43]. Previous studies have indicated that HCWs who report receiving adequate IPC training have a higher likelihood of complying with IPC than those who report inadequate or no training [44]. Our findings revealed that only among behavioural specialists, *perceived knowledge* and *perceived skills* were associated with IPC non-compliance. This can be attributed to differences in training and professional background. Knowledge and skills enhancing efforts such as education and training programmes may be less frequently aimed at non-medical professionals (e.g., behavioural specialists). In addition, non-medical professionals are often less aware of IPC procedures and tasks, and their role in preventing healthcare-associated infections [45].

This study’s finding that HCWs’ compliance with IPC is positively affected by the descriptive norm of the supervisor suggests that supervisors play an important role as role models for their subordinates. This is supported by previous studies in the nursing and hospital care setting, which have suggested that the presence of IPC role models is associated with increased IPC compliance among HCWs [17–19, 46]. The significance of the descriptive norm of the supervisor in our study, while the injunctive norm of the supervisor is not significant, may be explained by the fact that supervisors potentially have a strong social influence on their employees’ behaviour through their actions (descriptive norm), rather than through explicit directives or expectations (injunctive norm) [47]. This may reflect the influence of role modelling, whereby HCWs are more likely to adopt the behaviour of their supervisor as a positive role model, rather than as a result of feeling pressure to comply with their expectations.

Besides sociodemographic and psychosocial determinants of IPC compliance, IPC behaviour may be influenced by other factors, including environmental and logistical barriers, such as lack of necessary equipment as well as organisational culture, leadership, and policies [25, 27, 28, 39]. These barriers can make it difficult for HCWs to practice effective IPC, even if they have motivation, knowledge, and skills to do so. Although sociodemographic and psychosocial determinants are important factors for IPC compliance, the influence of other contextual factors, such as environmental and logistical barriers, should also be considered.

### Strengths and limitations

A strength of this study is the theoretical underpinning, as it uses concepts of multiple behaviour change theories. Previous studies have suggested the insufficiency of studies that used only one theory or a few theoretical models, and have highlighted that a broad theoretical underpinning enhances the quality of a study [46, 48]. Furthermore, the items included in our study were based on qualitative findings, thereby triangulating previous findings and providing additional insights into the topic [49].

This study has some limitations. Firstly, participants were selected by means of convenience sampling. This indicates that we cannot rule out some kind of selection bias, as perhaps, more IPC-minded HCWs were selected. Due to the recruitment method of participants (convenience sampling), it is challenging to accurately report the response rate, as the total number of professionals reached in the facilities is unknown. Nonetheless, we assume that the sample was rather representative of the study population since HCWs from different occupations (and therefore also different educational backgrounds) were reached. Secondly, the cross-sectional nature of the study precludes any causal inferences between compliance and the studied determinants [50]. One should note that there are also other potential psychosocial determinants of IPC compliance, besides the psychosocial determinants included in our study. An important determinant is risk perception, for which the perceived risk of infection (i.e., perceived susceptibility) for HCWs and their perceived severity are important components [51, 52]. Nevertheless, due to the length of the questionnaire and the required time investment of HCWs, we had to make choices about which determinants to include. Thirdly, one should take note of the cut-off point of 80% for sufficient compliance, as this may be too strict. Nevertheless, sensitivity analyses demonstrated that different operalisations of the cut-off point did not reveal other significant relevant results, therefore, indicating the robustness of our findings. In addition, due to the relatively small sample size in our study, it is likely that some associations were missed due to limited power, for example in the different categories of professional groups. For future studies, an increased sample size per professional group can provide an additional understanding of specific psychosocial determinants associated with IPC non-compliance, providing further insights for the design of improvement strategies and its targeting.

### Implications for practice

Our findings indicate the importance of increasing HCWs’ capabilities to comply with IPC. From the background of behaviour change theories, an initial recommendation for facilities is to implement structural education and training programmes. These educational efforts should not only include technical knowledge, but also aim to increase awareness, practical skills, and attitudes needed for proper IPC compliance, which can potentially increase HCWs’ capabilities. As the self-efficacy items *perceived inadequate education/training* and *perceived time constraints/competing priorities* were independently (of sociodemographic variables including professional group) associated with IPC non-compliance, there is a universal need among different HCWs for education and training programmes. Nevertheless, since professional group is a determinant of HCWs’ IPC compliance, educational efforts should be tailored to the educational needs of different professionals. Targeted educational strategies are especially important since our results indicated that the associations between *perceived knowledge and skills* and IPC non-compliance vary across different professional groups, with only behavioural specialists’ perceived knowledge and skills being associated with non-compliance. Therefore, we recommend directing education and training programmes towards the enhancement of knowledge and skills among behavioural specialists specifically.

The implementation of tailored education and training programmes can bridge the gap in IPC compliance between different HCWs. For example, non-medical professionals (e.g., behavioural specialists) may need basic IPC training and education to increase their awareness of IPC, whereas medical professionals may need more advanced IPC training to cover specialised areas of IPC. These educational efforts should be ongoing and continuous, as the effectiveness of these strategies may diminish over time [53]. Furthermore, educational programmes should incorporate an element that addresses perceived competing demands or time constraints. A potential strategy in this is professional-led education in which professionals come up with real-time examples and experiences in which they experienced competing demands or time constraints and discuss ways to overcome these challenges.

In addition to the importance of enhancing HCWs’ capabilities, the results of our study demonstrate the significant role of social norms − especially the descriptive norm of supervisors − on HCWs’ IPC compliance. Therefore, an additional strategy to promote IPC compliance includes role modelling and norm setting. We recommend that facilities raise awareness among supervisors about the impact of their role model function, and actively encourage them and other key individuals to model good IPC practices. This sets a positive example for HCWs, helps to increase the social norm for IPC compliance, and reinforces the importance of IPC, which helps to create a culture in the workplace and organisation in which IPC is emphasised and valued [17, 46]. Role models can be individuals who occupy formal leadership positions (i.e., supervisors), but they can also be informal leaders who influence the behaviour of others through their actions and attitudes.

Interventions regarding the use of role models have been implemented in the disability care sector in the Netherlands. On the initiative of the Limburg infection prevention and antibiotic resistance care network, some facilities started with the appointment of IPC contact persons. These individuals function as a first point of contact for their co-workers regarding IPC, act as a source of information, and play a stimulating role in raising awareness regarding the importance of IPC in the workplace.

Previous studies have suggested that behaviour change methods should be used in combination [54, 55]. For instance, norm setting should be used in conjunction with other intervention functions, such as education and training and role modelling, to maximise the effectiveness of the intervention. A strategy that may enhance both capabilities and facilitate IPC behaviour as a norm within the organisation is on-the-job training. On-the-job training could be a comprehensive approach, as it incorporates different elements, including the opportunity for HCWs to gain new skills and knowledge relevant to their job and IPC tasks, immediate feedback (also further enhancing capabilities), reinforcement of IPC norms, and a personalised (and tailored) approach to learning. On-the-job training makes the learning process more relevant and meaningful [56]; therefore, it potentially results in increased motivation among HCWs to comply with IPC. Evidence from previous studies has shown that IPC education and training involving HCWs in a practical, hands-on approach and incorporating individual experiences is associated with decreased healthcare-associated infections and increased IPC compliance [57, 58].

## Conclusions

Efforts to improve IPC compliance in the disability care setting should focus on strategies to enhance HCWs’ capabilities and decrease the gap in compliance between medical and non-medical professionals. A recommendation for facilities is to implement tailored and structural IPC education and training programmes, for which on-the-job training can be a relevant educational approach. In addition, the presence of role models − especially supervisors − is important to promote IPC behaviour. Facilities should create a culture of IPC compliance by norm setting, acting on, and modelling IPC behaviours at all levels of the organisation, including management, medical, and non-medical staff.

## Data Availability

The datasets used and analysed during the current study are available from the head of the data-archiving of the Public Health Service South Limburg on reasonable request. Interested researchers should contact the head of the data-archiving of the Public Health Service South Limburg (Tamara Kleine: tamara.kleine@ggdzl.nl) when they would like to re-use data.
